# Evaluation of reference genes for insect olfaction studies

**DOI:** 10.1186/s13071-015-0862-x

**Published:** 2015-04-22

**Authors:** Bonaventure Aman Omondi, Jose Manuel Latorre-Estivalis, Ivana Helena Rocha Oliveira, Rickard Ignell, Marcelo Gustavo Lorenzo

**Affiliations:** Chemical Ecology Unit, Department of Plant Protection Biology, SLU, Alnarp, Sweden; Current address: Bioversity International, Consultative Group for International Agricultural Research, Bujumbura, Burundi; Vector Behavior and Pathogen Interaction Group, Centro de Pesquisas René Rachou, FIOCRUZ, Belo Horizonte, Minas Gerais Brazil

**Keywords:** RT-qPCR, Reference genes, Normalization process, Olfaction and triatomines

## Abstract

**Background:**

Quantitative reverse transcription PCR (qRT-PCR) is a robust and accessible method to assay gene expression and to infer gene regulation. Being a chain of procedures, this technique is subject to systematic error due to biological and technical limitations mainly set by the starting material and downstream procedures. Thus, rigorous data normalization is critical to grant reliability and repeatability of gene expression quantification by qRT-PCR. A number of ‘housekeeping genes’, involved in basic cellular functions, have been commonly used as internal controls for this normalization process. However, these genes could themselves be regulated and must therefore be tested *a priori*.

**Methods:**

We evaluated eight potential reference genes for their stability as internal controls for RT-qPCR studies of olfactory gene expression in the antennae of *Rhodnius prolixus,* a Chagas disease vector. The set of genes included were: α-tubulin; β-actin; Glyceraldehyde-3-phosphate dehydrogenase; Eukaryotic initiation factor 1A; Glutathione-S-transferase; Serine protease; Succinate dehydrogenase; and Glucose-6-phosphate dehydrogenase. Five experimental conditions, including changes in age,developmental stage and feeding status were tested in both sexes.

**Results:**

We show that the evaluation of candidate reference genes is necessary for each combination of sex, tissue and physiological condition analyzed in order to avoid inconsistent results and conclusions. Although, Normfinder and geNorm software yielded different results between males and females, five genes (*SDH, Tub, GAPDH, Act* and *G6PDH*) appeared in the first positions in all rankings obtained. By using gene expression data of a single olfactory coreceptor gene as an example, we demonstrated the extent of changes expected using different internal standards.

**Conclusions:**

This work underlines the need for a rigorous selection of internal standards to grant the reliability of normalization processes in qRT-PCR studies. Furthermore, we show that particular physiological or developmental conditions require independent evaluation of a diverse set of potential reference genes.

**Electronic supplementary material:**

The online version of this article (doi:10.1186/s13071-015-0862-x) contains supplementary material, which is available to authorized users.

## Background

The kissing bug *Rhodnius prolixus* (Heteroptera; Reduviidae; Triatominae) is the second most important vector of Chagas disease, transmitting *Trypanosoma cruzi* to humans in Colombia and Venezuela [1-3]. This bug is considered a classical model for insect physiology and has been used extensively to study aspects of insect biology such as gut function [4,5], diuresis [6-10], neuropeptide production [11-14], and behaviour [15-17]. Moreover, *R. prolixus* has recently been suggested as a model to study the molecular bases of insect sensory perception and behaviour [18]. Quantitative reverse transcription PCR (qRT-PCR) or RNA-Seq analyses can be used to study these processes. However, a proper analysis based on these methods requires adequate normalization procedures.

Quantitative reverse transcription PCR (qRT-PCR) is the most robust and accessible method to detect and quantify messenger RNA (mRNA) transcription levels associated with specific physiological conditions [19-22] or genetic manipulation, e.g. RNA interference [23-25]. The technique has also been used to validate RNA-Seq [26-28] and microarray results [29]. Quantitative reverse transcription PCR offers sensitivity, a large dynamic range, accurate quantification, and the possibility to measure expression levels in different samples for a number of target genes [21,30-32]. It also enables intensive replication and statistical analysis that is not possible with more expensive methods like RNA-Seq. However, qRT-PCR is subject to systematic error from biological and technical limitations set by: (i) use of samples with different quality and amount of starting material, which affects RNA extraction efficiency; (ii) inaccurate quantification of the extracted RNA; (iii) variability of the reverse transcription reaction efficiency; and (iv) highly subjective data analysis [20,21,30-33]. These limitations make the systematic normalization of gene expression data important to improve the fidelity and accuracy of qRT-PCR. For this, the expression levels of a target and a reference gene are simultaneously measured in a sample. Subsequently, any changes in target gene expression are expressed relative to those of the reference gene, a process named relative quantification. An alternative is to refer target gene expression to sample concentration, also called absolute quantification. The first is the ideal choice when one intends to compare the relative change in expression of a target gene under different physiological conditions. In contrast, absolute quantification is more adequate when the total number of copies of a gene is to be determined.

For relative quantification, an ideal reference gene should have minimal expression variability in the different tissues, cells or physiological states under study [20-22,30-38]. Intuitively, this may imply that genes like α-tubulin, *GAPDH* or β-actin that are required for structural and basic cellular function (housekeeping genes) would be best suited for this purpose. However, stably expressed genes may not exist in practice [30,31]. In fact, some of the most common reference genes used to date have been shown to suffer significant regulation under specific experimental conditions, and are therefore inappropriate for normalization purposes [39-45]. For this reason, an ideal approach would be to test a set of potential reference genes in all experimental conditions to be studied and select the best-suited genes for each comparison [19,30,31,33]. Nevertheless, many gene expression studies use reference genes without previously validating expression stability [21,46]. In case the expression profile of the selected reference gene is altered between the experimental conditions being studied, this change would be transferred to the calculations made on the dataset, biasing the conclusions obtained [20-22,30-33]. For *R. prolixus*, the stability of several reference genes has already been tested in different tissues (anterior and posterior midgut, ovary, fat body, flight muscle and salivary glands) considering the effects of nutrition and trypanosome infection [38,47]. Nevertheless, further studies dealing with different tissues and experimental conditions in this species are required to avoid basing the choice of reference genes exclusively on previous studies.

Transcript levels are generally low in studies dealing with tissues expressing olfactory genes. Furthermore, changes in olfactory gene expression under different physiological conditions are often subtle and, therefore the normalization of expression data is a critical process. The olfactory system is crucial for insects, and it plays a fundamental role in host location by insect vectors [48-50]. qRT-PCR has been widely used for the functional characterization of many genes related to olfaction in different insects such as *Drosophila* [51], mosquitoes [52,53], moths [54,55], bees [56,57], and termites [58]. However, few studies have presented evidence of objective selection of reference genes for the tissues and conditions under study.

The present work analyzed the stability of the expression profiles of eight potential reference genes in the antennae of *R. prolixus*. These profiles were compared separately in both sexes for five different conditions involving changes in nutritional status, developmental stage and adult age.

## Methods

### Insects

Insects were obtained from the colony of intradomiciliary *R. prolixus* established in Centro de Pesquisas René Rachou (CPqRR) more than 20 years ago (donated by Dr. Carlos Ponce, Ministerio de Salud Pública, Honduras). Experimental insects were reared under controlled conditions at 26 ± 1°C, 65 ± 10.0% relative humidity, and a 12:12 h light/dark illumination cycle provided by artificial lights (4 fluorescent lamps, cold white light, 6400K, 40 W). All tests were performed with 5^th^ instar larvae or adults, separately for both sexes. All ages subsequently described for treatments represent the number of days elapsed after insects underwent ecdysis to their current instar. The expression of reference gene candidates was analyzed in five different conditions: (i) 21 day-old unfed 5^th^ instar larvae; (ii) 21 day-old fed 5^th^ instar larvae; (iii) 1 day-old unfed adults; (iv) 21 day-old unfed adults; (v) 21 day-old fed adults. An artificial feeder was used five days before sample preparation to feed the corresponding insects with citrated rabbit blood (2.5% buffered sodium citrate) provided by the Centro de Criação de Animais de Laboratório (CECAL) from Fundação Oswaldo Cruz (FIOCRUZ). Six samples of 60 antennae each (i.e., from 30 bugs) were analyzed for each of the five treatments.

### Candidate reference genes and primer design

Eight candidate reference genes previously used for qRT-PCR normalization in triatomines [38,47] and other insect species [19,33,59,60] were selected: α-tubulin (*Tub*); β-actin (*Act*); Glyceraldehyde-3-phosphate dehydrogenase (*GAPDH*); Eukaryotic initiation factor 1A (*eIF-1a*); Glutathione-S-transferase (*GST*); Serine protease (*Sp*); Succinate dehydrogenase (*SDH*); and Glucose-6-phosphate dehydrogenase (*G6PDH*). All of these genes were identified in the *R. prolixus* genome database (available in www.vectorbase.org/organisms/rhodnius-prolixus) using a local tBLASTn algorithm [61] and orthologous sequences from UniProt Knowledgebase (details showed in Additional file 1: Table S1), except for *Sp* already identified in *R. prolixus* by Bedoya and Lowenberger (Sequence ID: B8QQQ1, submitted in April 2008 to EMBL/GenBank/DDBJ databases). Primers were designed using Primer3 4.0.0 (http://primer3.wi.mit.edu) [62]. The melting temperature was set at 60°C and the specificity for each primer was tested *in silico* using BLASTn [63] in the *R. prolixus* genome database. As much as possible, primers were designed to amplify a product between 100 to 200 bp, to flank or straddle an intron, proximate to the first exons at the 3’ terminus of each gene, to promote amplification efficiency, and exclude amplification of genomic DNA. The primer pairs were tested for homo- and hetero-dimerisation using the Oligoanalyser online tool (Integrated DNA Technologies, Inc. IA, USA). The main characteristics of the designed primers are shown in Table 1.

**Table 1:** **Characteristics of the candidate reference genes and ionotropic receptor co-receptor primers**

**Gene**	**Biological function**	**Primer sequence (5’to 3’)**	**Amplicon length (bp)**	**Intron length (bp)**	**R** ^**2**^	**E (%)**
*Act*	Cytoskeletal protein	For- TGTCTCCCACACTGTACCCATCTA /	87	338	0.992	88.2%
		Rev- TCGGTAAGATCACGACCAGCCAA				
*eIF-1a*	Protein biosynthesis	For- TTGGAGGCCATGTGCTTTGAT /	94	183	0.999	91,3%
		Rev-AGGTTTCTTGCTTCATCTGGAGT				
*GAPDH*	Glycolytic protein	For- GACTGGCATGGCATTCAGAGTT /	182	1130	0.992	102.5%
		Rev- CCCCATTAAAGTCCGATGACACC				
*GST*	Metabolism	For- TACCCATCATTTGGCGTGGACA /	177	Intron - Exon junction	0.987	103.2%
		Rev- CAAACCCAATTGCCTCAGCGAT				
*G6PDH*	Metabolism	For- AGCCTGGAGAAGCGGTTTACGTTA /	162	923	0.998	96.5%
		Rev- GTGAGCCACAGAATACGTCGAGT				
*SDH*	Metabolism	For- TTGCCGGAGTAGATGTTACCAG /	147	1592	0.999	104.8%
		Rev- CAGCTGCATAAAGTCCTTCCAC				
*Sp*	Metabolism	For- AGGGACCATCTTTGACTGCTCTTC/	157	Intron - Exon junction	0.996	98.8%
		Rev- GAATCACCCTGGCAAGCATCTTTT				
*Tub*	Structural subunit of microtubules	For- TGTGCCCAAGGATGTGAACG/	118	202	0.991	110.9%
		Rev- CACAGTGGGTGGTTGGTAGTTGAT				
*RproIR76b*	Ionotropic receptor co-receptor	For- GCGTTTGCGTACCAAATGGACA /	113	1055	0.974	84.1%
		Rev- GCGTCCGGTAGATCCAAAGTGATT				

Biological function; primer sequences; amplicon and intron lengths, R^2^: squared correlation coefficient (calculated from the regression line of the standard curve); E: quantitative real-time PCR efficiency (calculated by the standard method).

### RNA extraction and cDNA synthesis

Total RNA was extracted in 500 μL of TRIzol® Reagent (Life Technologies, Carlsbad, CA, USA) from pools of 60 antennae according to the manufacturer’s instructions. The extracted RNA was resuspended in 30 μL of DEPC-treated water (Life Technologies). RNA concentration was determined using a Qubit® 2.0 Fluorometer (Life Technologies) and RNA integrity was analyzed by means of electrophoresis in 2% agarose gels visualized after GelRed™ staining (Biotium Inc, Hayward, CA, USA). Genomic DNA was eliminated using the RQ1 RNase-Free DNase kit (Promega, Fitchburg, WI, USA). All treated RNA (11 μL) was immediately used to synthesize cDNA samples using the SuperScript III Reverse Transcriptase (Life Technologies) and a 1:1 mix of Random Hexamers and 10 μM Oligo (dT) 20 primers in a final volume of 20 μL. The reverse transcription reactions were performed in a MasterCycler ® Gradient Thermal Cycler (Hauppauge, NY, USA) under the following conditions: 10 min at 25°C; 60 min at 50°C and 15 min at 70°C. Finally, cDNAs were stored at −20°C.

### Quantitative real-time PCR

For qRT-PCR, 10 μL of SYBR Green PCR Master Mix® (Life Technologies) were used for reaction mixtures that also contained 1 μL of 2-fold diluted cDNA sample and 0.8 μL of a 10 μM primer solution in a final reaction volume of 20 μL. The reactions were performed in an ABI PRISM 7500 Sequence Detection System (Life Technologies) under the following conditions: 10 min at 95°C, followed by 40 cycles of 15 s at 95°C, 20 s at 60°C, and 30 s at 72°C. After the amplification step, melting curve analyses (HRM rate = 0.5°C), and electrophoreses in 2% agarose gels visualized after GelRed™ staining (Biotium Inc, Hayward, CA, USA) were performed with qRT-PCR products to confirm reaction specificity. Reactions for each sample were performed in three technical replicates. In all qRT-PCR experiments, no-template controls (NTC) were included in triplicates. PCR efficiencies (E) for each primer were determined using the slope of a linear regression model [35], which was obtained by measuring the quantification cycle (Cq) for a range of 5-fold serial dilutions of cDNA samples (except for *Act* that was obtained using a 3-fold serial dilution). Information about primers, amplicons and calibration curves is presented in Table 1.

### RT-PCR and sequencing

The PCR reactions for the eight reference genes were performed for 35 cycles (94°C for 30 s, 60°C for 30 s and 72°C for 30 s) with 2 μL of pure cDNA, 2.2 μL of a 1 mM dNTP solution, 0.6 μL of a 10 μM primer solution, and 1 U of Taq polymerase (Promega) in a final volume reaction of 20 μL. Expected amplicon sizes in PCR products were confirmed using 2% agarose gels visualized after GelRed^TM^ staining (Biotium Inc, Hayward, CA, USA). Afterward, PCR products were purified using the Wizard Genomic DNA Purification Kit (Promega) and sequenced using both primers with the ABI Prism BigDye V 3.1 Terminator Cycle Sequencing kit and an ABI 3730 DNA sequencing system (Life Technologies). The consensus sequences were obtained using the Staden Package 2.0 [64] and verified by comparing them with the *R. prolixus* genomic database, using the basic local alignment search tool (BLASTn).

### Data analysis

#### Ranking candidate reference genes

The geNorm [21] and Normfinder [65] algorithms were used to detect the most stable reference genes for each comparison using the GenEx software v. 5.4 (MultiD Analyses AB, Sweden). geNorm estimates the gene expression stability measure (M-value) calculating the mean pairwise variation (V) of each gene relative to all other genes included in the analysis. Genes with the lowest M-value are considered the most stable genes in the tested conditions. In calculation, at each step, the least stable gene is eliminated and an M-value is recalculated until the two most stable genes are defined. Normfinder estimates the expression stability (reported as expression stability value or SV) in and between groups using a two-way ANOVA. According to Normfinder, genes with the lowest SV are the most stable. In our analysis, three different stability rankings were obtained, one from geNorm (based on M-value) and two from Normfinder (based on SV). Of these, one considered experimental groups (hereafter named as Normfinder^W^), while the other omitted these groups (hereafter named as Normfinder).

Normfinder also allowed estimating the optimal number of reference genes needed for normalization by calculating the accumulated standard deviation (Acc. SD) based on any number of reference genes. Therefore, the number of genes showing the lowest Acc. SD was used to determine how many genes to select to build normalization factors (NFs) for each comparison intended. This was estimated separately for female and male antennae for all comparisons studied. This algorithm also indicated the best pairwise combination of most stable genes. It is worth noting here that the mentioned combination was only used in those cases in which the optimal number of reference genes suggested by Normfinder was two.

#### Normalization factor selection

The NFs were created and evaluated for each condition following different steps. First, the optimal number of reference genes defined for each comparison was used to select the best genes according to geNorm and both Normfinder rankings. Subsequently, the geometric mean of the selected genes was calculated to create the corresponding NFs. Then, all NFs were ranked together with the single genes using the geNorm and Normfinder algorithms. Finally, the NF placed in best position after considering the three rankings was selected. In case of a tie in the latter step, both alternatives were tested to confirm that they did not alter the overall result.

The analysis of expression stability of candidate reference genes, the calculation of the optimal number of references genes and the selection of the NFs were performed to evaluate results separately for the antennae of males or females in 8 pairwise comparisons (four *per* sex): (i) 21 day-old unfed larvae and 21-day old fed larvae (effect of nutrition in larval bugs); (ii) 21 day-old unfed adults and 21-day old fed adults (effect of nutrition in adults); (iii) 21-day old unfed larvae compared to 1 day-old unfed adults (effect of moulting); (iv) 1 day-old unfed adults compared to 21 day-old unfed adults (effect of maturation in adults).

#### Normalization process over different approaches

To highlight the influence of the normalization process on the analysis of gene expression data from qRT-PCR, the transcript abundance for an olfactory co-receptor gene (*RproIR76b*) was analyzed in the conditions earlier described using two different normalization approaches. The *RproIR76b* dataset used for this purpose has been generated (and has been already used) for the purpose of studying molecular aspects of triatomine bug olfaction and is here used to exemplify the impact of reference gene selection on normalization processes (Latorre-Estivalis et al., submitted). First, qRT-PCR data were normalized using the best reference gene or the best normalization factor obtained as previously explained. In the second approach, the normalization was performed using the least stable candidate reference gene according to the rankings generated by geNorm, Normfinder and Normfinder^W^. For each treatment, fold-change values were subjected to statistical analysis to estimate the influence of data treatment on the expression profile of the olfactory receptor gene. Data were analyzed using the Kolmogorov–Smirnov test to check normality using the Graph-Pad Prism® (5.0) software. Data not satisfying normality assumptions were Log transformed. Finally, a two-tailed *t*-test for pair-wise comparisons was performed considering probabilities of *p* < 0.05 as significant.

## Results

### Expression profiles of candidate reference genes

**Figure 1:**
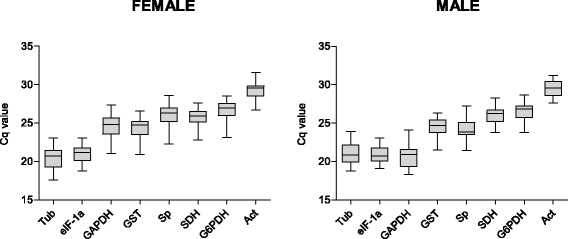
Expression levels of candidate reference genes in female and male antennae of *R. prolixus*. Grey bars indicate the 25/75 percentiles, whisker caps indicate the 10/90 percentiles, and the line marks means. Cq: quantitative cycle.

The expression of the eight reference genes in *R. prolixus* antennae was initially confirmed by the presence of a single band of the expected size in 2% electrophoresis agarose gels. Amplicon sequencing confirmed the specificity and correct design of all primers. The presence of a single-peak in the dissociation curve obtained at the final step of qRT-PCR also validated primer specificity. The qRT-PCR efficiency and determination coefficient (R^2^) of each gene are detailed on Table 1. Reaction efficiency varied from 88.2% (*Act*) and 110.9% (*Tub*) and the regression coefficients ranged between 0.999 (*SDH*) to 0.987 (*GST*). The raw quantification cycle (Cq) values were situated between 20.4 (*Tub*) and 29.3 (*Act*) in female antennae and between 20.9 (*eIF-1a*) and 29.5 (*Act*) in male antennae (Figure 1).

### Analysis of gene expression stability

All algorithms previously mentioned were used to calculate gene expression stability for the eight candidate reference genes in all comparisons. Candidate reference genes were ranked separately for females (Table 2) and males (Table 3) from the most to the least stable considering their average expression stabilities (M-values from geNorm) and their expression stability values (SVs from Normfinder and Normfinder^W^).

**Table 2:** **Gene expression stability rankings for the different physiological conditions studied in female antennae**

**Ranking position**	**Effect of nutrition in larvae**			**Effect of nutrition in adults**			**Imaginal moult effect**			**Adult maturation effect**		
	**geNorm**	**NormFinder**	**NormFinder** ^**w**^	**geNorm**	**NormFinder**	**NormFinder** ^**w**^	**geNorm**	**NormFinder**	**NormFinder** ^**w**^	**geNorm**	**NormFinder**	**NormFinder** ^**w**^
1	Tub/GAPDH	Tub	Tub	G6PDH/GAPDH	Tub	Tub	G6PDH/SDH	SDH	SDH	Sp/GAPDH	Tub	Tub
	(0.215)	(0.079)	(0.083)	(0.327)	(0.186)	(0.169)	(0.387)	(0.193)	(0202)	(0.345)	(0.271)	(0.268)
2	-	SDH	G6PDH	-	G6PDH	SDH	-	GAPDH	GAPDH	-	G6PDH	G6PDH
		(0.155)	(0.125)		(0.256)	(0.232)		(0.217)	(0.208)		(0.284)	(0.326)
3	GST	GST	GST	Tub	SDH	G6PDH	GAPDH	G6PDH	Act	G6PDH	SDH	SDH
	(0.251)	(0.171)	(0.133)	(0.352)	(0.269)	(0.253)	(0.476)	(0.431)	(0.323)	(0.393)	(0.3)	(0.326)
4	G6PDH	G6PDH	SDH	eIF-1a	GAPDH	GAPDH	Act	Act	G6PDH	Tub	Sp	GST
	(0.272)	(0.2)	(0.158)	(0.388)	(0.331)	(0.261)	(0.543)	(0.492)	(0.478)	(0.42)	(0.515)	(0.403)
5	SDH	GAPDH	GAPDH	SDH	eIF-1a	eIF-1a	Sp	Sp	Sp	SDH	GST	Sp
	(0.301)	(0.268)	(0.18)	(0.407)	(0.35)	(0.267)	(0.578)	(0.607)	(0.570)	(0.515)	(0.533)	(0.51)
6	eIF-1a	eIF-1a	Act	Sp	Sp	Sp	GST	Tub	Tub	GST	GAPDH	GAPDH
	(0.348)	(0.494)	(0.306)	(0.444)	(0.474)	(0.288)	(0.629)	(0.704)	(0.692)	(0.56)	(0.568)	(0.548)
7	Act	Act	eIF-1a	GST	GST	GST	Tub	GST	GST	eIF-1a	eIF-1a	eIF-1a
	(0.406)	(0.502)	(0.333)	(0.485)	(0.508)	(0.305)	(0.708)	(0.823)	(0.811)	(0.618)	(0.589)	(0.559)
8	Sp	Sp	Sp	Act	Act	Act	eIF-1a	eIF-1a	eIF-1a	Act	Act	Act
	(0.523)	(0.888)	(0.395)	(0.529)	(0.575)	(0.345)	(0.799)	(0.997)	(0.955)	(0.736)	(1.052)	(0.902)
			Best combination			Best combination			Best combination			Best combination
			GP6PDH–GST			G6PDH–eIF-1a			GAPDH–SDH			G6PDH–SDH
			(0.068)			(0.101)			(0.077)			(0.079)

geNorm and Normfinder rankings were built using M-value and stability value, respectively. The Normfinder ranking did not take experimental groups into account, while the Normfinder^w^ one considered them for the analysis of expression stability.

**Table 3:** **Gene expression stability rankings for the different physiological conditions studied in male antennae**

**Ranking** **position**	**Effect of nutrition in larvae**			**Effect of nutrition in adults**			**Imaginal moult effect**			**Adult maturation effect**		
	**geNorm**	**NormFinder**	**NormFinder** ^**w**^	**geNorm**	**NormFinder**	**NormFinder** ^**w**^	**geNorm**	**NormFinder**	**NormFinder** ^**w**^	**geNorm**	**NormFinder**	**NormFinder** ^**w**^
1	G6PDH/GAPDH	SDH	G6PDH	SDH/Tub	SDH	SDH	SDH/GAPDH	Act	SDH	GST/G6PDH	G6PDH	Sp
	(0.14)	(0.051)	(0.141)	(0.306)	(0.259)	(0.155)	(0.332)	(0.423)	(0.283)	(0.378)	(0.421)	(0.296)
2		G6PDH	SDH		GAPDH	Act	-	GAPDH	GAPDH	-	Sp	GST
		(0.128)	(0.164)	-	(0.265)	(0.195)		(0.424)	(0.293)		(0.447)	(0.334)
3	Tub	GAPDH	GAPDH	GAPDH	Tub	G6PDH	Act	G6PDH	Sp	eIF-1a	Tub	G6PDH
	(0.215)	(0.162)	(0.18)	(0.361)	(0.298)	(0.212)	(0.419)	(0.426)	(0.313)	(0.494)	(0.452)	(0.391)
4	SDH	Tub	Tub	Act	Act	Tub	G6PHD	SDH	Act	Sp	eIF-1a	SDH
	(0.227)	(0.195)	(0.224)	(0.39)	(0.331)	(0.214)	(0.536)	(0.432)	(0.410)	(0.564)	(0.454)	(0.399)
5	GST	GST	GST	G6PDH	G6PDH	GAPDH	GST	Sp	G6PDH	Tub	GST	Tub
	(0.319)	(0.35)	(0.344)	(0.423)	(0.375)	(0.244)	(0.578)	(0.492)	(0.426)	(0.599)	(0.511)	(0.411)
6	Act	Act	Sp	GST	GST	GST	Sp	Tub	Tub	SDH	SDH	eIF-1a
	(0.399)	(0.628)	(0.499)	(0.484)	(0.504)	(0.246)	(0.615)	(0.544)	(0.528)	(0.659)	(0.527)	(0.429)
7	eIF-1a	eIF-1a	Act	eIF-1a	eIF-1a	eIF-1a	Tub	GST	GST	Act	Act	GAPDH
	(0.486)	(0.697)	(0.577)	(0.52)	(0.519)	(0.325)	(0.665)	(0.631)	(0.617)	(0.687)	(0.634)	(0.543)
8	Sp	Sp	eIF-1a	Sp	Sp	Sp	eIF-1a	eIF-1a	eIF-1a	GAPDH	GAPDH	Act
	(0.578)	(0.8)	(0.636)	(0.598)	(0.779)	(0.35)	(0.726)	(0.806)	(0.767)	(0.715)	(0.64)	(0.571)
			Best combination			Best combination			Best combination			Best combination
			SDH–GST			Act–SDH			Tub–GST			G6PDH–eIF-1a
			(0.126)			(0.114)			(0.149)			(0.156)

geNorm and Normfinder rankings were built using M-value and stability value, respectively. The Normfinder ranking did not take experimental groups into account, while the Normfinder^w^ one considered them for the analysis of expression stability.

#### The effect of nutrition

In all three rankings obtained for antennae of unfed and fed female larvae *Tub* was the most stable gene, while *Sp* was the least stable one (Table 2). The five most stable genes for this comparison (*Tub, GAPDH, GST, G6PDH* and *SDH*) and three least stable genes (*eIF-1a, Act* and *Sp*) were the same in the three rankings generated. In this case, the combination of *G6PDH*-*GST* was suggested as the best option by Normfinder^W^.

All genes had a stable expression profile when unfed and fed male larvae antennae were compared, as the highest M and SV values were 0.59 and 0.80, respectively (Table 3). In this case, the most stable gene was *G6PDH*, which was ranked first by geNorm and Normfinder^W^, while *eIF-1a*, *Act* and *Sp* were again ranked in the last positions. For this comparison the best combination suggested by Normfinder^W^ was *SDH-GST*.

As with female larvae, the comparison of expression patterns from antennae of unfed and fed female adults showed that *Tub* was the most stable gene (Table 2). All candidate reference genes were very stable between these conditions, with highest M and SV values of 0.53 and 0.57, respectively (Table 2). Similarly as with female larvae, *eIF-1a*, *Sp* and *Act* were characterized as unstable genes, although *GST* was also unstable in female adults. The two rankings obtained with Normfinder were identical except for the position of *G6PDH* and *SDH*. Despite *eIF-1a* was not ranked in top positions in any of the rankings, this gene together with *G6PDH* were considered as the best combination by Normfinder^W^.

Feeding male adults did not seem to affect the stability of this set of genes, and *SDH* was identified as the most stable gene (Table 3). In fact, all genes showed good expression stability (maximum M-value = 0.59 and maximum SV = 0.77). In this comparison geNorm and both Normfinder rankings were identical except for the *GAPDH* and *Tub*. The *GST*, *eIF-1a* and *Sp* genes were characterized as the worst by geNorm and both Normfinder rankings. Curiously and contrasting the three previous comparisons, *Act* was not ranked in the lowest four ranking positions. Interestingly, the expression levels of *eIF-1a* and *Sp* genes were the most unstable when the antennae of larval and adult males were compared.

#### The effect of imaginal moult

In the comparison of unfed 21 day-old female larvae against 1-day old adult females, the stability of all candidate genes decreased as the highest M and SV values were 0.79 and 0.99, respectively (Table 2). For the corresponding comparison in males M (0.72) and SV (0.80) values were slightly lower (Table 3). Coincidently, the comparison of the effect of development on gene expression in female and male antennae showed *SDH* and *GAPDH* as the most stable genes. On the other hand, *Sp*, *Tub*, *GST* and *eIF-1a* were ranked in the last four positions both in female and male rankings.

#### The effect of adult maturation

Female maturation during the first phase of imaginal life (1 day-old *vs* 21-day-old) affected *Tub* and *G6PDH* the least (Table 2). M-values varied from 0.35 (*Sp* and *GAPDH*) to 0.73 (*Act*), while SV-values ranged from 0.27 to 1.05 in Normfinder and from 0.26 to 0.9 in Normfinder^W^. Except for the order of *GST* and *Sp*, both Normfinder rankings were identical in this case. *eIF-1a* and *Act* genes were considered as the least stable genes taking into account the results from the three rankings. Normfinder^W^ proposed the combination of *G6PDH* and *SDH* as the most stable for comparing the effect of imaginal maturation in female antennae.

The rankings generated by geNorm and Normfinder when 1 day-old and 21 day-old males were compared were quite different and only the positions of *Act* and *GAPDH* as the least stable genes matched all classifications (Table 3). The analysis suggested *G6PDH* as the most stable gene for this comparison due to the fact that it was ranked first in two of the rankings.

### Calculation of the optimal number of reference genes for data normalization

**Figure 2:**
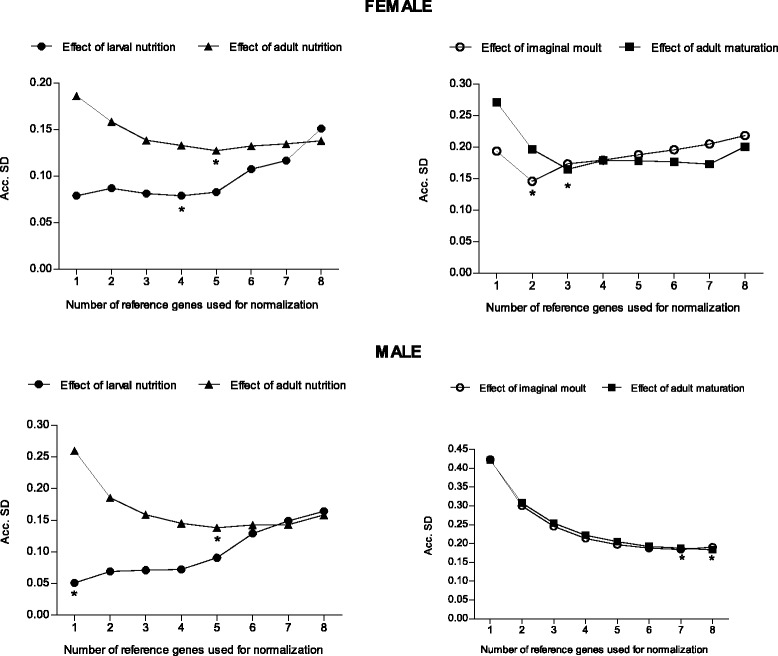
Optimal number of reference genes calculated by Normfinder for normalization in female and male antennae of *R. prolixus*. Asterisks mark the number of reference genes (the lowest Acc. SD value) used for normalization in each condition.

The number of genes which when combined showed the lowest accumulated standard deviation (Acc. SD) was selected for each experimental condition studied using geNorm (Figure 2). The optimal number of genes to be used in normalization procedures for the five different treatments ranged from only one, up to eight (i.e., all genes studied). Four genes were enough to normalize expression values in antennae from unfed and fed female larvae. In antennae from male larvae, this normalization could be performed with only one gene. For both sexes, the optimal number of reference genes to normalize expression data from antennae of unfed and fed adults was five. Regarding moulting and developmental effects on female antennae, the ideal number of genes to normalize gene expression values were two and three, respectively. In contrast, the comparison of gene expression on male antennae from unfed larvae and 1-day old adults would require combining the seven most stable genes. Finally, to analyze the developmental effect in male adults, all 8 potential reference genes would be necessary for an optimal normalization process.

### Normalization factor construction

The elaboration of NFs for the various conditions analyzed in female antennae was laborious. In the experiment about the effect of adult nutrition, the combination of *G6PDH*-*GAPDH*-*Tub-eIF-1a* and *SDH* was selected (see Table 4). For the other experiments with females, the absence of consensus between different rankings made additional comparisons necessary. In these cases, different NFs were created and subsequently compared in an iterative manner (Table 4). In the case of the experiment on larval nutrition, the number of genes recommended by Normfinder was four, and two different NFs were created: one (*Tub-GST-G6PDH-GAPDH*) based on the geNorm stability ranking and another (*Tub-GST-G6PDH-SDH*) based on the Normfinder stability rankings described in Table 2. Both NFs showed high stability (M-value = 0.1 and maximum SV = 0.06) and they were ranked first in the three new rankings. The result of the rankings made it impossible to select a NF between the two better ranked for comparing nutrition effects on antennae of female larvae. Therefore, normalization was performed using both NFs separately and the results obtained were compared to confirm that the different NFs ranked as best did not alter the conclusions reached in this case. For the experiment testing the effect of development to the adult phase in female antennae, two combinations, including the best pairs of genes from geNorm (*G6PDH-SDH*) and Normfinder (*G6PDH-SDH*), were also very stable (Table 4). When ranked in a second round, the Normfinder combination appeared as best in all rankings. Finally, two different factors with three different genes were generated for the maturation experiment on adult female antennae: *G6PDH-Sp-GAPDH* (geNorm) and *G6PDH-SDH-Tub* (Normfinder and Normfinder^W^), the latter being the most stable (Table 4). The NFs chosen for the different experiments on the antennae of females are described in Table 5.

**Table 4:** **Stability values of the female normalization factors**

**Effect of nutrition in larvae**			**Imaginal moult effect**			**Adult maturation effect**		
**geNorm**	**NormFinder**	**NormFinder** ^**w**^	**geNorm**	**NormFinder**	**NormFinder** ^**w**^	**geNorm**	**NormFinder**	**NormFinder** ^**w**^
**Tub-GST-**	**Tub-GST-**	**Tub-GST-**						
**G6PDH-GAPDH**	**G6PDH-GAPDH**	**G6PDH-GAPDH**	**SDH-GAPDH**	**SDH-GAPDH**	**SDH-GAPDH**	G6PDH-Sp-GAPDH	G6PDH-Sp-GAPDH	G6PDH-Sp-GAPDH
(0.103)	(0.047)	(0.065)	(0.241)	(0.193)	(0.076)	(0.257)	(0.35)	(0.366)
Tub-GST-	Tub-GST-	Tub-GST-	G6PDH-SDH	G6PDH-SDH	G6PDH-SDH	**G6PDH-Tub-SDH**	**G6PDH-Tub-SDH**	**G6PDH-Tub-SDH**
G6PDH-SDH	G6PDH-SDH	G6PDH-SDH	(0.191)	(0.217)	(0.251)	(0.303)	(0.116)	(0.038)
(0.103)	(0.0516)	(0.055)						
		Best gen			Best gen			Best gen
		Tub-GST-						
		G6PDH-SDH			SDH-GAPDH			G6PDH-Tub-SDH
		(0.055)			(0.076)			(0.038)

The Normfinder ranking did not take experimental groups into account, while the Normfinder^w^ one considered them for the analysis of expression stability. The normalization factor selected is shown in bold for each of the five comparisons.

**Table 5:** **Genes used for building the best normalization factors for each experimental comparison**

	**Effect of nutrition in larvae**	**Effect of nutrition in adults**	**Imaginal moult effect**	**Adult maturation effect**
Female antennae	Tub-GAPDH-GST-G6PDH or Tub-GAPDH-GST-SDH	G6PDH-GAPDH-Tub-eIF-1a-SDH	SDH-GAPDH	G6PDH-Tub-SDH
Male antennae	G6PDH	SDH-Tub-GAPDH-Act-G6PDH	All genes except eIF-1a	All genes

For comparing the effect of feeding on gene expression in the antennae of female larvae it was impossible to select only one NF and both best ranked gene associations were included in the table.

The *G6PDH* gene, ranked best in all three rankings (Table 3) would be the choice to normalize expression data from the nutrition experiment with male larvae. The combination of *SDH-Tub-GAPDH-Act* and *G6PDH* (these five genes appear in the first positions in all rankings, see Table 3) would be the choice to normalize expression data from the nutrition experiment performed with male adult antennae. To compare gene expression levels in antennae of larval and recently emerged adult males, all genes except for *eIF-1a* (see Table 3) would be needed. Besides, the combination of all genes would be required to compare gene expression data between antennae of 1 and 21 day-old male adults. All NFs selected for males are detailed on Table 5.

### Verifying the effect of different normalization strategies on the expression profile calculated for a target gene

**Figure 3:**
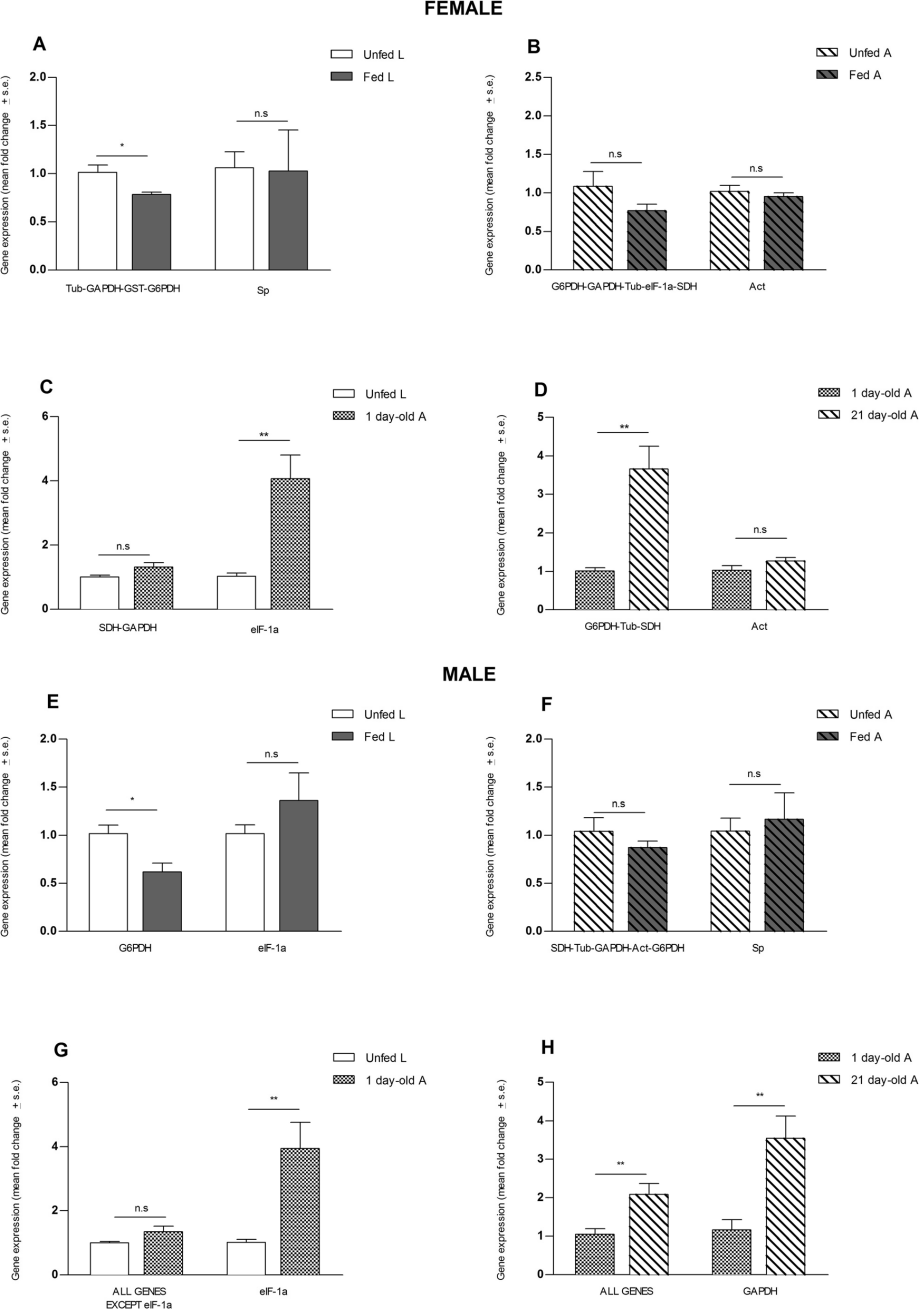
Relative expression of *RproIR76b* in female and male antennae of *R. prolixus* using different normalization approaches. Two normalization approaches were used: applying the best normalization factor (left) and the worst potential reference gene (right). **(A)** Effect of nutrition in antennae of female larvae; **(B)** Effect of nutrition in antennae of female adults; **(C)** Effect of imaginal moulting in female antennae; **(D)** Effect of adult maturation in antennae of female adults; **(E)** Effect of nutrition in antennae of male larvae; **(F)** Effect of nutrition in antennae of male adults; **(G)** Effect of imaginal moulting in male antennae; and **(H)** Effect of adult maturation in antennae of male adults. Significant differences were calculated by using a two-tailed *t*-test for pair-wise comparisons. Asterisks denote statistically significant differences, * p <0.01; ** p < 0.01 and *** p < 0.0001. Error bars represent the standard error generated from 6 replicates *per* condition. L: larvae; A: adult; n.s.: non-significant difference.

Two different approaches were used to evaluate the expression levels of an olfactory co-receptor gene in the antennae of female and male bugs for the comparisons listed above. In a first case, normalization was performed with the NFs described in Table 4. Alternatively, the same process was performed using the least stable gene selected according to the stability rankings calculated (see Tables 2 and 3). Interestingly, in only three of the eight comparisons performed, both normalization approaches produced identical results, i.e., with both adult nutrition experiments (Figure 3b and f) and male adult maturation (Figure 3h). For all other comparisons, the conclusions about stability, increase or decrease on gene expression were altered, such as with female larvae nutrition (Figure 3a) or female adult maturation (Figure 3d).

## Discussion

A current objective of diverse insect olfaction studies is to understand the molecular basis of olfactory detection, by associating transcription profiles with physiological conditions. The qRT-PCR technique has been widely used in these studies, and to validate molecular techniques such as RNA interference [66] or RNA-Seq [27,67]. In this study, a set of eight candidate reference genes was evaluated in the antennae of an insect vector in five different conditions for both sexes. The importance of using a suitable normalization approach in olfactory gene studies based on qRT-PCR was shown.

In qRT-PCR experiments, testing potential reference genes enables the use of the most stable internal controls from a panel of candidate genes. A number of ‘housekeeping genes’ involved in basic cellular functions, such as energy production or cell division, have been commonly used reference genes. Many of these genes work well for RT-PCR where end point determination is done, and are useful candidates in qRT-PCR. However, the expression of these genes may be regulated under certain physiological comparisons; e.g. the expression of *GAPDH* has been shown to be subject to regulation in intestinal tissues after blood ingestion in triatomines [38]. For this reason, evaluating a set of candidate reference genes is necessary for each combination of species, tissues, sex and physiological conditions tested in order to avoid inconsistent results. Considering the geNorm and Normfinder stability rankings and the normalization factors (NFs) created in our study, the most stable genes in *Rhodnius* antennae were *G6PDH*, *Tub*, *GAPDH* and *SDH*. In fact, *SDH* was ranked in the top positions in all the comparisons, except for fed male larvae. *Tub* has already been reported as a stable gene in salivary glands and crops of *R. prolixus* [38].

The use of an inappropriate normalization protocol impacts directly on the interpretation of results, as shown by the analysis of *RproIR76b* expression. A potential impact could be a misleading statistical result, an increased variation between replicates or even a reversal of the regulation direction reported. The importance of this aspect is reflected by the example used in our study, where transcriptional signal changes depended on the stability of the reference genes chosen. For example, there were no significant differences in expression between larvae and adults from both sexes using the combination of the most stable genes. Instead a clear, but false, increase in *RproIR76b* expression was generated when qRT-PCR data were normalized using an unstable gene as *eIF-1a* (Figure 3). A similar situation was observed with male larvae when data were normalized using *eIF-1a*; according to these calculations, feeding induced a false up-regulation of *RproIR76b* expression, while normalization with the most stable gene combination resulted in an opposite effect (Figure 3). Majerowicz et al. [47] observed that *eIF-1a* was useful to normalize gene expression data from different tissues, such as the posterior midgut, ovary and fat body. In the same study, *Act* was identified as a stable gene in the ovaries. This last candidate is one of the first genes proposed as a reference for normalization in qRT-PCR [68]. In contrast, we observed that the least stable genes in *R. prolixus* antennae were *Sp, eIF-1a* and *Act*. Our study demonstrates that reference gene stability can change between physiological conditions, tissues and sexes within the same species, showing the limitations of adopting previously used reference genes in qPCR experiments.

The number of genes used to create the normalization factors is also a critical point in the normalization process. Although we selected a number of genes as most reliable references, it is still possible that other genes not tested would emerge as more stable for these comparisons. For instance, even with a panel of eight potential reference candidates, all genes were necessary to create the most stable normalization factor in the experiment studying the effect of male development. This highlights the importance of evaluating a relevant number of genes to allow a proper normalization process to find the most stable combination of genes available [32].

The use of reference gene selection software was not a straightforward process in our study. Particularly, the different softwares generated stability rankings that after comparing them, evinced several inconsistencies. It would be desirable that the different approaches end up with the same genes ranked as best candidates, especially when targeting multiple treatments and comparisons. However, a consensus of the best gene or genes was not always possible. One problem in selecting reference genes observed with the software we used was that stability indicators of a single gene are always tied to those of the other genes considered in the same panel and to the elimination method used. geNorm uses an elimination approach to arrive at the most stable pair of genes based on cumulative standard deviation, while Normfinder uses pairwise stability of single or selected groups of genes. Used alone, Normfinder might therefore find two co-regulated genes as most stable combination. However across analysis software, reference factors comprising of more than one reference gene always emerged more stable than the single genes alone. Therefore considering a batch of the most stable individual genes together to design a reference factor provides a more reliable means of normalization irrespective of method of choice of the internal calibrator.

## Conclusions

This study underlines the need for an appropriate selection of internal normalization factors (and genes) for qPCR studies. We showed that, for genes with slight regulation, accurate normalization could, in fact change, the regulation signal obtained. This study further reiterates that while some traditionally used genes are an important starting point, new studies in specific tissues and treatment conditions need to objectively validate them for those conditions. For olfactory genes, such validation is much more important given the low expression levels of many genes in this system making the genes especially sensitive to regulatory changes acting on genes used as reference factors. The genome of *R. prolixus* will be published soon and is expected to lead to transcriptomics studies by qRT-PCR or RNA-Seq. Olfaction has also become a focus of studies looking after behavioural modification of pests and vector species [50]. Insect olfaction is known to be plastic and often regulated by the physiological needs of a species. Together, these facts would be expected to result in a rise in genomic studies of olfaction, and the attendant need for rigorous normalization protocols. This study is therefore an important step, and to our knowledge the first focusing on normalization procedures for the study of olfactory genes of insect vector species.

## Additional file

Additional file 1: Table S1. Candidate reference genes, orthologous sequences used for tBLASTn searches, supercontig location of candidate genes in the *R. prolixus* genome, VectorBase gene codes and number of exons and number of amino-acids.

## Abbreviations

qRT-PCR: Quantitative reverse transcription polymerase chain reaction
mRNA: messenger RNA
*Tub*: α-tubulin
*Act*: β-actin
*GAPDH*: Glyceraldehyde-3-phosphate dehydrogenase
*eIF-1a*: Eukaryotic initiation factor 1A
*GST*: Glutathione-S-transferase
*Sp*: Serine protease
*SDH*: Succinate dehydrogenase
*G6PDH*: Glucose-6-phosphate dehydrogenase
bp: Base pairs
HRM: High resolution melt
NTC: Non-template controls
Cq: Quantification cycle
M-value: Gene expression stability measure
V: Pairwise variation
SV: Stability value
ANOVA: Analysis of variance
Normfinder^W^: Normfinder without treatments
Acc. SD: Accumulated standard deviation
NFs: Normalization factors
*RproIR76b*: *Rhodnius prolixus* ionotropic receptor 76b
