# Supplementary material for: Evaluation of reference genes for insect olfaction studies
Source: Parasit Vectors. 2015 Apr 22;8:243. doi: 10.1186/s13071-015-0862-x (PMC4417234; doi:10.1186/s13071-015-0862-x)
Supplement: Additional file 1: Table S1. — Candidate reference genes, orthologous sequences used for tBLASTn searches, supercontig location of candidate genes in the R. prolixus genome, VectorBase gene codes and number of exons and number of amino-acids. [file 13071_2015_862_MOESM1_ESM.docx]

Table s1. Candidate reference genes, orthologous sequences used for tBLASTn searches, supercontig location of candidate genes in the *R. prolixus* genome, VectorBase gene codes, number of exons and number of amino-acids.

| **Gene** | **Orthologous sequences** | ***R. prolixus* genome**  **Supercontig location** | **Gene Code** | **N° of exons** | **Length (amino-acids)** |
| --- | --- | --- | --- | --- | --- |
| *Act* | E0VKP4 (*P. humanus*);  Q6PPI5 (*H. vitripennis*);  B0XB41 (*C. quinquefasciatus*);  P10981 (*D. melanogaster*) | GL561372 | RPRC008015 | 3 | 374 |
| *eIF-1a* | A6YPT9 (*T. infestans*);  E0VEI0 (*P. humanus*);  B0X9F4 (*C. quinquefasciatus*);  Q9VEA1 (*D. melanogaster*) | GL563067 | RPRC013802 | 5 | 146 |
| *GAPDH* | D6WV03 (*T. castaneum*);  E0VE33 (*P. humanus*);  B0WEB5 (*C. quinquefasciatus*);  P07486 (*D. melanogaster*) | GL563035 | - | 7 | 333 |
| *GST* | I6TU46 (*A. gossypii*);  Q9113 (*An. gambiae*);  Q9VG92 (*D. melanogaster* | GL562715 | RPRC013179 | 5 | 219 |
| *G6PDH* | D6WKK9 (*T. castaneum*);  B0WHG8 (C*. quinquefasciatus*);  P12646 (*D. melanogaster*) | GL563007 | RPRC012854 | 9 | 519 |
| *SDH* | E0VZC3 (*P. humanus*);  B0WFX7 (C*. quinquefasciatus*);  Q94523 (*D. melanogaster*) | GL563067 | RPRC013824 | 14 | 667 |
| *Sp* | B8QQQ1 (*R. prolixus*) | GL562567 | RPRC003090 | 8 | 381 |
| *Tub* | Q964U9 (*C. tentans*);  E0VSM7 (*P. humanus*);  B0XE76 (*C. quinquefasciatus*);  D6WBN7 (*T. castaneum);*  P06603 (*D. melanogaster*) | GL562849 | RPRC003672 | 4 | 449 |
